# Characterization of a Novel Fe^2+^ Activated Non-Blue Laccase from *Methylobacterium extorquens*

**DOI:** 10.3390/ijms23179804

**Published:** 2022-08-29

**Authors:** Abidan Ainiwaer, Yue Liang, Xiao Ye, Renjun Gao

**Affiliations:** Key Laboratory for Molecular Enzymology and Engineering, The Ministry of Education, School of Life Science, Jilin University, Changchun 130012, China

**Keywords:** laccase, *Methylobacterium extorquens*, characterization, Fe^2+^ ion

## Abstract

Herein, a novel laccase gene, *Melac13220*, was amplified from *Methylobacterium extorquens* and successfully expressed in *Escherichia coli* with a molecular weight of approximately 50 kDa. The purified Melac13220 had no absorption peak at 610 nm and remained silent within electron paramagnetic resonance spectra, suggesting that Melac13220 belongs to the non-blue laccase group. Both inductively coupled plasma spectroscopy/optical emission spectrometry (ICP-OES) and isothermal titration calorimetry (ITC) indicated that one molecule of Melac13220 can interact with two iron ions. Furthermore, the optimal temperature of Melac13220 was 65 °C. It also showed a high thermolability, and its half-life at 65 °C was 80 min. Melac13220 showed a very good acid environment tolerance; its optimal pH was 1.5. Cu^2+^ and Co^2+^ can slightly increase enzyme activity, whereas Fe^2+^ could increase Melac13220′s activity five-fold. Differential scanning calorimetry (DSC) indicated that Fe^2+^ could also stabilize Melac13220. Unlike most laccases, Melac13220 can efficiently decolorize Congo Red and Indigo Carmine dyes even in the absence of a redox mediator. Thus, the non-blue laccase from *Methylobacterium extorquens* shows potential application value and may be valuable for environmental protection, especially in the degradation of dyes at low pH.

## 1. Introduction

With the rapid development of global industrialization, water pollution has become a common problem in the past few years. Synthetic dye wastewater from the textile industry is regarded as the main source of water pollution. It is estimated that 2–20% of the annual output of industrial textile dyes is directly discharged into natural water bodies in the form of wastewater [1]. Traditional dye treatment methods include chemical and physical methods, including coagulation, adsorption, and oxidation. However, due to the high cost of operation and raw materials associated with these methods, they are unsuitable for the industrial scale. In recent years, as awareness of environmental protection has increased, many industries have been striving to develop green chemical technology via enzymatic approaches. This is largely due to the increasing attention being paid to environmental issues, relevant legal restrictions, and an increased understanding of laccases. The outstanding biochemical properties of laccase enzymes have made them more useful to the scientific community [2,3,4].

Common laccases (EC 1.10.3.2, benzenediol: oxygen oxidoreductases) are blue multi-copper-containing polyphenol oxidases because laccases typically contain four copper ions which are divided into three copper centers, type one (T1), two (T2), and three (T3), according to their spectroscopic properties. Among these, type one (T1) copper shows an electron absorption band around 610 nm, which is why the laccase is blue. Type two (T2) copper has no absorption in the visible spectrum but has an EPR signal, whereas type three (T3) copper is an antiferromagnetic coupling ion, showing electron adsorption around 330 nm. Like other enzymes widely found in nature, including terrestrial and marine sources [5,6], laccases are also an extraordinary group of metalloenzymes that are widely distributed around us. Speaking of marine enzymes, since the oceans cover 70% of the Earth’s surface, it has a wider range of biodiversity than the land. Therefore, special attention should be paid to the potential of marine enzymes. The marine-derived laccase also showed unique enzymatic properties [7]. Recently, laccases have also been found in human epithelial cells [8]. Although most of the laccases in nature conform to the descriptions above, some laccases display the absence of the characteristic adsorption spectrum for T1 Cu, and these are endowed with some atypical characteristics, such as stability and oxidation capacity [9]. These laccases are known as non-blue laccases. Laccases have broad substrate specificity and can catalyze the oxidation of many organic compounds, such as phenols, chlorophenols, aromatic and aliphatic amines, and some inorganic compounds [10,11]. Since first being used in industry in the 1990s, laccases have been widely employed in diverse biotechnological applications, e.g., bioremediation, textile dye decolorization, food industries, organic synthesis, and biofuel production [10,12,13,14,15]. Although laccases have a wide range of sources, laccases derived from plants and animals have not reached a suitable yield. Because of the higher redox potential of fungal laccases compared to bacterial laccases (470–810 mV vs. 400 mV) [16], the laccase from fungi have been the focus of research, and the current industrial laccases mainly originate from fungal sources. However, a long fermentation period and poor stability in high-temperature and high-salinity environments have limited their further applications. Bacterial laccases, by contrast, have gained more attention because they lack glycosylation, have a shorter fermentation period, are easy to extract, and their purification has good potential for future applications. Furthermore, they have several advantages over fungal laccases, including a wider optimal pH range, higher thermostability, and greater tolerance to alkaline environments [17,18]. In addition, with the help of redox mediators, bacterial laccases can obtain a higher redox potential to degrade refractory substrates. Therefore, bacterial laccases may be promising alternatives to fungal laccases in some specific industrial applications.

The main disadvantage of fungal laccase is its instability at high temperatures. Therefore, the production of laccase is currently aimed at the exploration of thermophilic bacteria. The discovery of thermophilic bacteria will be conducive to the development of thermophilic enzymes for industrial applications. In general, thermophilic enzymes have several advantages over mesophilic enzymes, including resistance to chemical denaturants, high alkalinity and extreme acidity, higher reaction rates, less susceptibility to microbial contamination, and better substrate diffusion [19]. Generally, the temperature of the wastewater released from the dyeing process is always above 50 °C [20]. Therefore, the ability of laccases to operate at higher temperatures is particularly important since elevated temperatures often favor high reaction rates in many processes, which means higher decolorization velocity. In order to avoid the additional cooling process to reduce the cost and make full use of the high temperature of dyeing wastewater to achieve the maximum decolorization rate in a short time, laccases with high optimal temperature and excellent thermal stability are required. The aim of the present study was to find a novel bacterial laccase that exhibits high activity at a high temperature and to further biochemically characterize its expression and purification in *E. coli*. In the present study, we report the first non-blue laccase derived from *Methylobacterium extorquens*, providing its detailed characterization and describing its heterologous expression in *E. coli* and its purification with SP cationic exchange chromatography. Additionally, the recombinant enzyme with unique spectral properties and metal content was characterized and tested in dye decolorization and degradation processes. Given the versatility of laccase, the isolation and characterization of this novel non-blue bacterial laccase and the exploration of its useful industrial properties will be helpful in enhancing its application in industry or environmental pollution treatments, especially in some low-pH environments.

## 2. Results

### 2.1. Recombinant Expression and Purification of Melac13220

**Figure 1:**
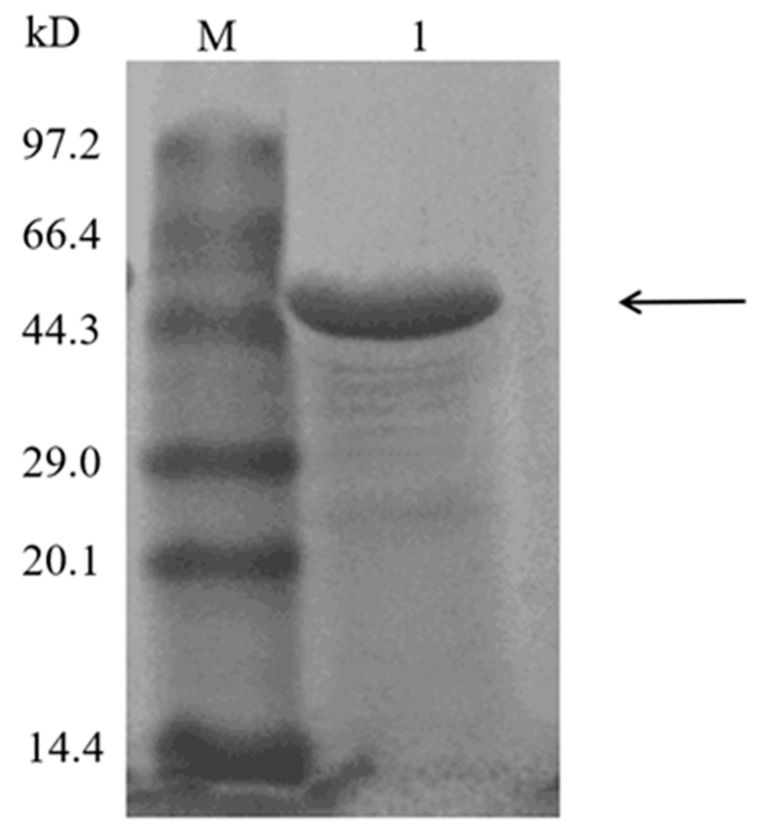
Expression and analysis of the molecular weight of Melac13220 via SDS-PAGE using SP cationic exchange column. Lane M: molecular weight marker; Lane 1: purified Melac13220. The band of Melac13220 is marked with black arrow.

The gene encoding laccase Melac13220 from *Methylobacterium extorquens* was cloned into the expression vector pET-28a (+) and transformed into *E. coli* BL21 (DE3) to express recombinant enzyme. The recombinant protein was purified by SP cationic exchange column. The elution buffer with NaCl eluted a single target protein band, and laccase was determined to be 51-fold enriched with a recovery of 72%, and the yield of recombinant protein was 30 mg/L. Compared with the molecular weight marker (Figure 1), Melac13220 had the predicted molecular weight of 50 kDa with a single protein band on SDS-PAGE as visualized by Coomassie Brilliant Blue staining, which is similar to other bacterial laccases [21,22]. The specific activity values of crude and purified Melac13220 with ABTS as a substrate were 21.87 U/mg (7.238 mg/mL) and 515.52 U/mg (0.625 mg/mL), respectively. Indicating that the purification reached 23.6-fold.

### 2.2. Effect of Temperature and pH on the Activity and Stability of Melac13220

**Figure 2:**
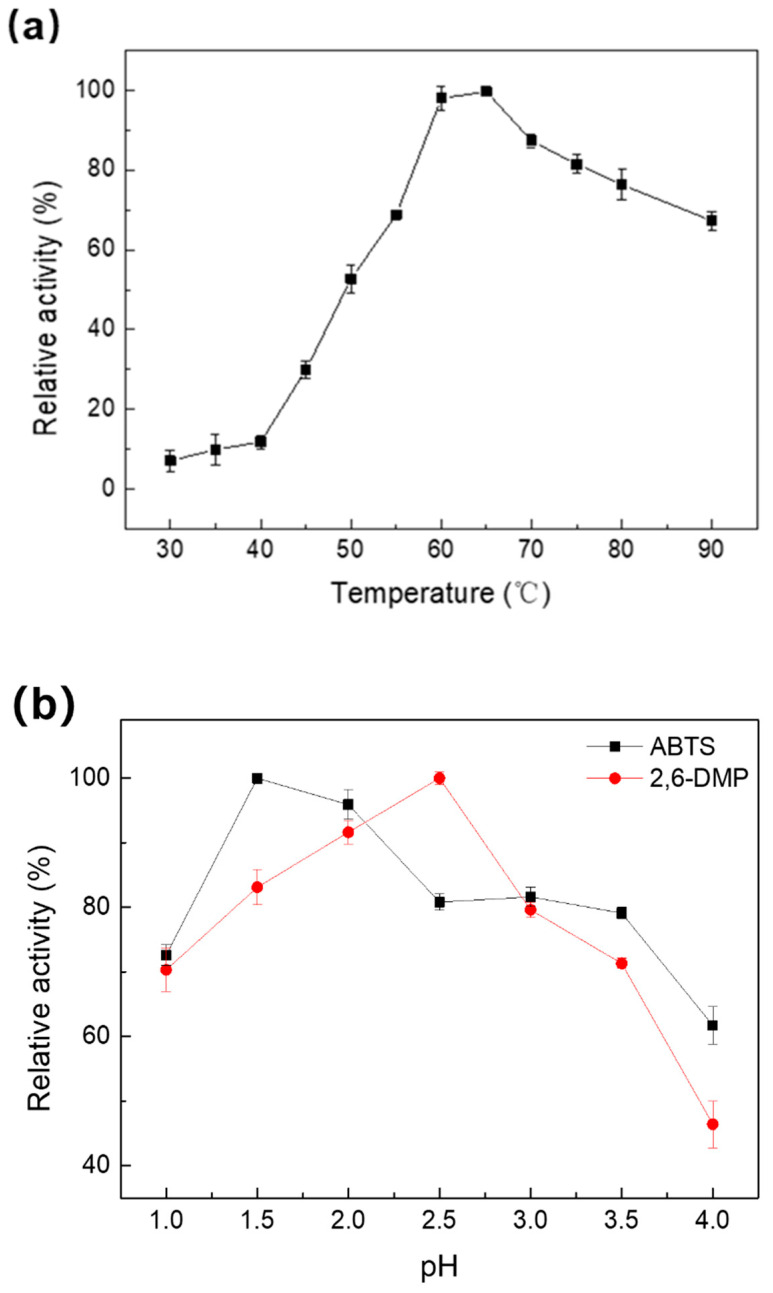
Characterization of purified recombinant Melac13220 from *Methylobacterium extorquens*; (**a**) optimal temperature in reaction with ABTS as a substrate; (**b**) optimal pH in reaction with ABTS and 2,6-DMP. All experiments were performed in triplicate, and the results represent mean ± standard deviation.

The performance of Melac13220 was studied at different temperatures. Melac13220 was highly active toward ABTS in the temperature range of 60 °C to 90 °C (Figure 2a). The maximum activity was defined as 100% relative activity. The laccase activity linearly increased between 40 °C and 60 °C. Purified laccase showed the maximum enzyme activity at 65 °C (Figure 2a), but it also worked efficiently at temperatures of 70 °C to 80 °C. Therefore, Melac13220 maintained good catalytic activity at high temperatures. In contrast, the optimum temperatures for most fungal-derived laccases are lower than those of bacterial laccases [23,24,25,26]. When the temperature reached 90 °C, the activity of Melac13220 rapidly declined. The enzyme activity increased with temperature and displayed the highest activity when oxidizing ABTS at 65 °C, but it declined above 70 °C.

Generally, laccases have different optimum pH depending on the substrate. The activity of Melac13220 towards ABTS and 2,6-DMP was only reported at a narrow pH range of approximately 1.0 to 4.0 (Figure 2b) and decreased with increasing pH values. The pH activity profile was similar to a bell-shaped curve, indicating that purified Melac13220 is a typical bacterial laccase and is more active in extremely acidic conditions. The highest enzyme activities were recorded at pH 1.5 for ABTS and pH 2.5 for 2,6-DMP, which were much lower than those reported for bacterial or fungal laccases. Moreover, the optimum pH of 1.5 was by far the lowest optimum pH value. Moreover, beyond pH 4.0, enzyme activity was severely reduced, and the enzyme maintained less than 60% of its original activity. Therefore, it is reasonable to speculate that this laccase is an acidic-tolerant enzyme.

**Figure 3:**
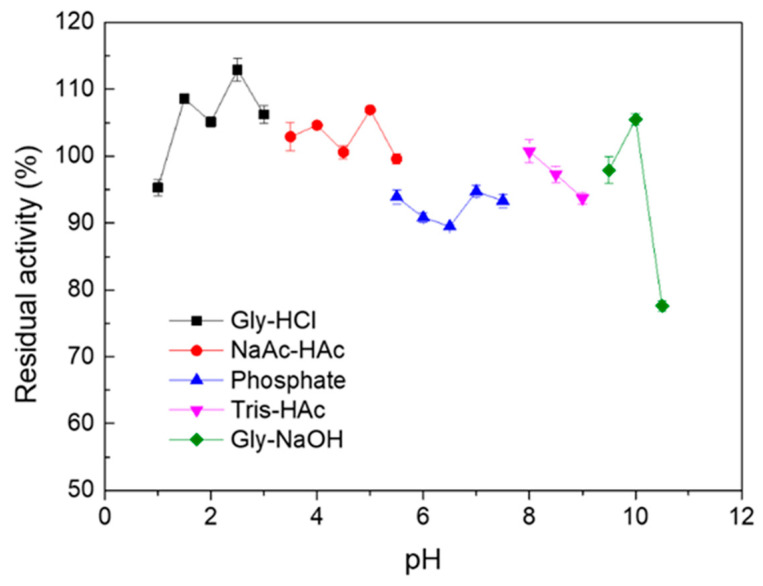
The pH stability of Melac13220 was assayed at 50 °C after the incubation of laccase in different pH levels buffers for 1 h using ABTS as substrate. All experiments were conducted 3 times, and the results represent mean ± standard deviation.

Melac13220 retained more than 75% of its laccase activity within the pH range of 1.0 to 10.5 after incubation for 1 h (Figure 3). This revealed that Melac13220 showed pH-stable conformational states in the evaluated pH range. Although its stability decreased slightly at pH values over 10, it remained at a high level of residual activity. For this reason, Melac13220 demonstrated high stability in a wide pH range. 

**Figure 4:**
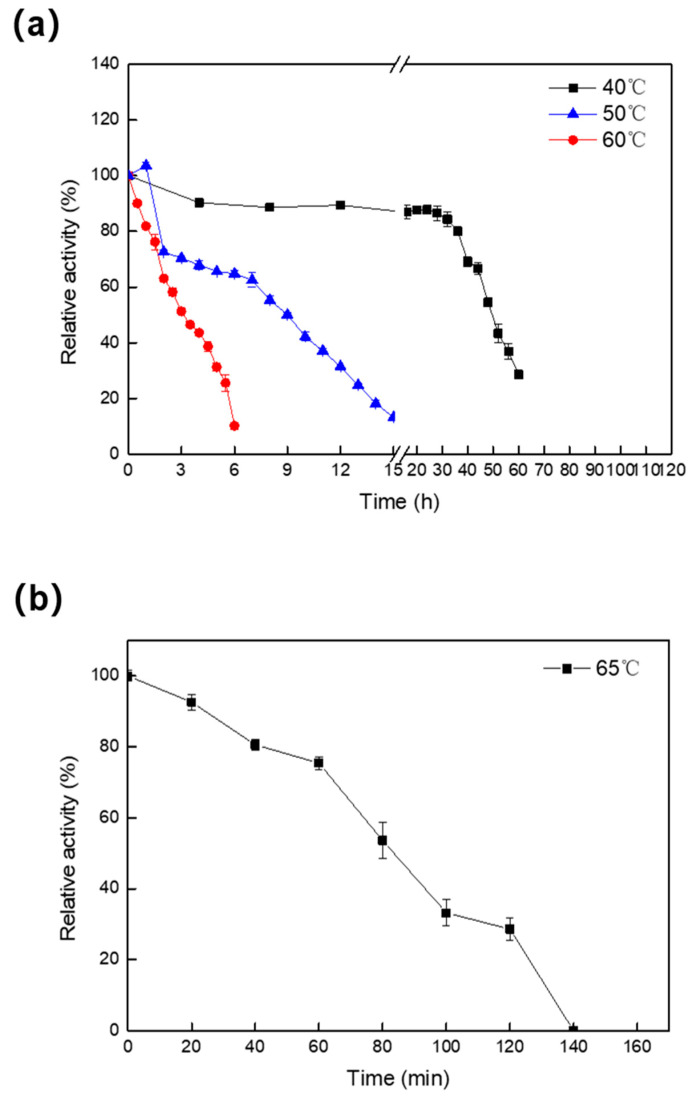
The thermostability of Melac13220 after incubation of the purified laccase at 40, 50 °C, 60 °C (**a**) and 65 °C (**b**) with different durations. All the results represented as the means ± standard deviation from at least three independent experiments.

The thermal stability of Melac13220 (Figure 4) was calculated by assessing residual enzyme activity after incubation at varying intervals at 40 °C, 50 °C, 60 °C, and 65 °C. As laccase was relatively stable at 40 °C, the activity of laccase was measured every 4 h. As shown in the figure, the activity of laccase decreased slowly and was more stable at 40 °C than at 50 °C. The half-life of Melac13220 at 40 °C and 50 °C was 50 h and 9 h, respectively. The increased activity in the first 1 h was probably due to the transient thermal activation effect of the enzyme. In order to determine the thermal stability of the enzyme at 60 °C, samples were taken every 0.5 h to measure the residual activity. The thermal stability of the enzyme at 60 °C was far less than that at 40 °C and 50 °C. However, the enzyme activity dropped sharply with a half-life of approximately 3 h, and it maintained approximately only 8% of the initial activity after 6 h of incubation. The thermal stability of the laccase at 65 °C is shown in Figure 4b. The laccase was completely inactivated at 140 min, and the activity dropped rapidly within 60 min, with a half-life of approximately 80 min. In summary, the enzyme maintained excellent stability over 40 °C with a half-life of 50 h. After incubation for 7 h, Melac13220 retained about 60% of the original activity at 50 °C, which means this enzyme can be placed in the set of moderately thermostable enzymes. However, the stability of the enzyme is poor at high temperatures, which has also been reported in other studies [26].

In order to measure protein unfolding temperature, differential scanning calorimetry (DSC) provides unique information about the thermal stability of proteins based on their thermal variation [27]. The melting temperature (T_m_) of purified Melac13220 was 57 ± 0.2 °C, and the enthalpy of melting (ΔH_l_) was (2.29 ± 0.06) × 10^5^ kcal/mol (Supplementary Figure S1a). This indicates that melting occurred at temperatures above 55 °C. After adding 1 mM of Cu^2+^ and Fe^2+^, the melting temperature of the enzyme rose above 60 °C (Supplementary Figure S1b,c). The thermodynamic stability of the enzyme can be evaluated using this parameter. The high melting temperature is a good indication of the thermal stability of Melac13220, which was consistent with the optimum temperature of the enzyme.

### 2.3. Effect of Metal Ions and Organic Solvent on the Activity of Melac13220

**Figure 5:**
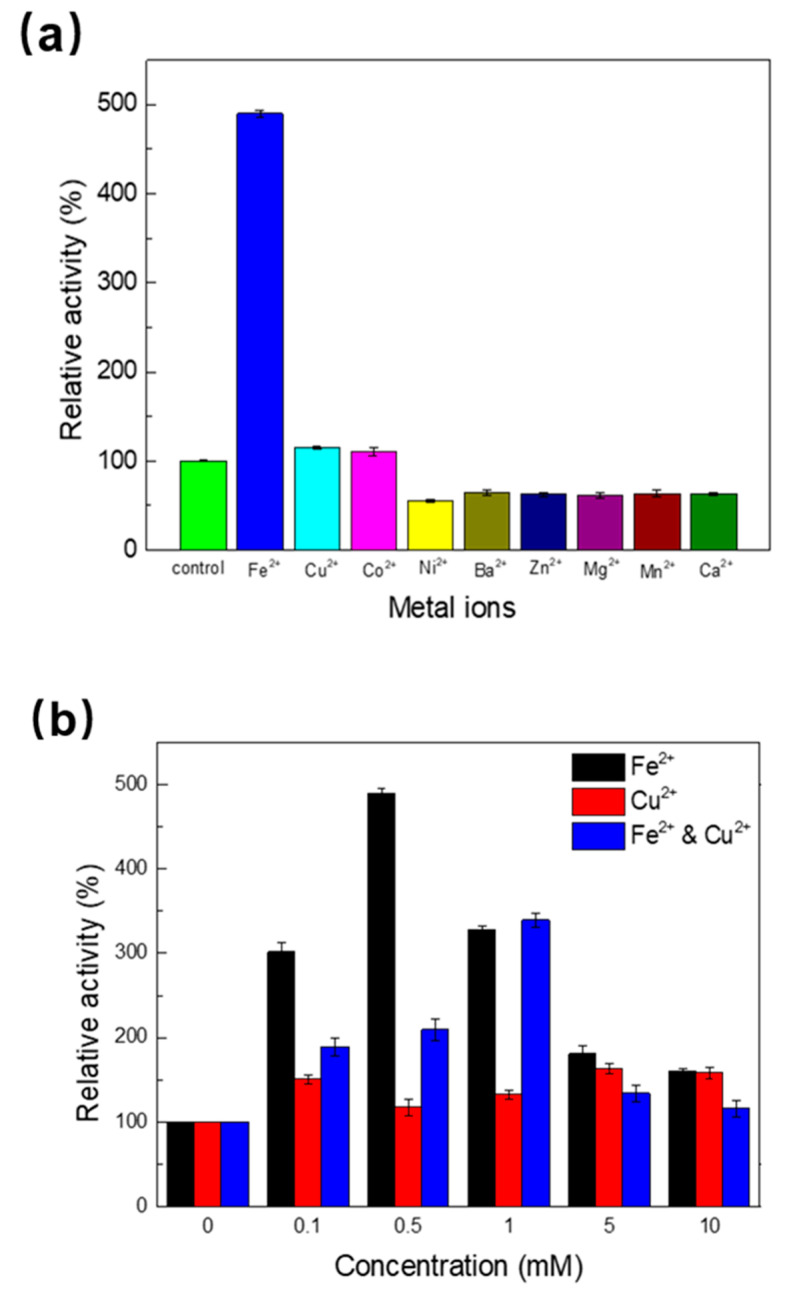
Activity of Melac13220 after 1 h of pre-incubation in the presence of various metallic ions in the reaction mixture (**a**) and at 0.1 mM, 0.5 mM, 1 mM, 5 mM and 10 mM concentration of Fe^2+^, and Cu^2+^ (**b**). The enzyme activity in the absence of metal ions was regarded as 100%. The data represented as the mean ± standard deviation from at least independent experiments.

The activity of laccase against different exogenous divalent metal ions (0.5 mM) was determined (Figure 5a). Among the metals tested, enzyme activity was promoted by Fe^2+^, Cu^2+^, and Co^2+^, whereas Ni^2+^, Ba^2+^, Zn^2+^, Mg^2+^, Mn^2+^, and Ca^2+^ inhibited laccase activity. Fe^2+^ remarkably promoted laccase activity, which was inconsistent with the findings of others [28,29,30]. Cu^2+^ and Co^2+^ slightly enhanced the activity of laccase, as previously reported [28,29,31]. The effect of Fe^2+^ and Cu^2+^ on enhanced enzyme activity appeared to be concentration-dependent (Figure 5b). This is shown by the enhancement of Melac13220 laccase activity under different concentrations of Fe^2+^ and Cu^2+^, especially in the presence of 0.5 mM Fe^2+^, which increased enzyme activity five-fold. Thus, Fe^2+^ was the best activator of Melac13220 among all the tested metals. For Cu^2+^, the increase in enzyme activity was greatest when the final concentration reached 5 mM. Baldrian and Gabriel also concluded that Cu^2+^ positively affected laccase activity [32].

**Figure 6:**
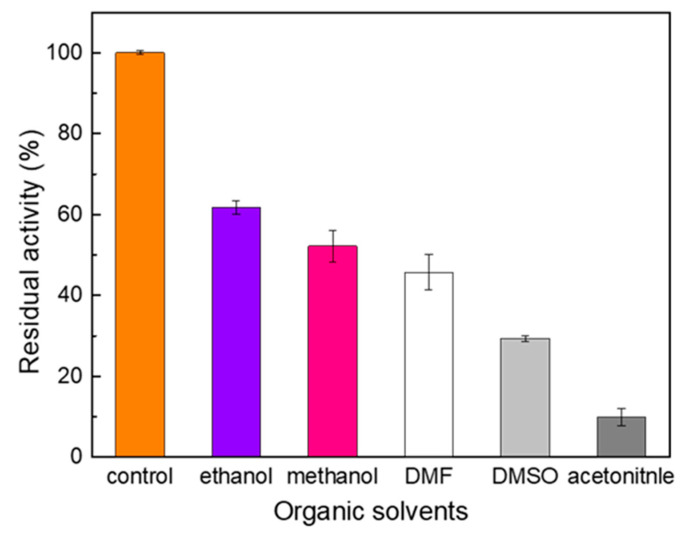
Activity of Melac13220 after 1 h of pre-incubation in the presence of organic solvents at 10% (*v*/*v*) concentration. The enzyme activity in the absence of organic solvents was regarded as 100%. Values represent the mean ± standard deviation of three measurements.

Organic solvents are the most commonly used non-aqueous media for biocatalysts [33]. Therefore, a better understanding of the activity profile of laccases in organic solvents can contribute to identifying the most suitable reaction media for a given biocatalyst. The influence of various organic solvents on Melac13220 activity was evaluated with ABTS as substrate, and the results are shown in Figure 6. Enzyme activity was inhibited by all the organic solvents (10%(*v*/*v*)) tested, among which ethanol had the least influence, with its activity remaining at 60%. Acetonitrile had the greatest inhibitory effect, with only approximately 10% of the enzyme activity remaining after 1 h of incubation.

### 2.4. Enzyme Kinetic Properties

The kinetic constants of Melac13220 were obtained with ABTS and 2,6-DMP as substrates at the optimum pH for the enzyme based on the Michaelis–Menten equation via non-linear regression. These results may be due to their different active site geometries to accommodate the substrate [24]. The K_m_, k_cat_, and k_cat_/K_m_ values of Melac13220 for ABTS and 2,6-DMP, as well as the results of the addition of Fe^2+^, are shown in Table 1. These results indicate that Melac13220 had a greater affinity for ABTS than 2,6-DMP and demonstrate that it had much better substrate affinity than other bacterial laccases. The kinetic constants of other bacterial laccases with ABTS as a substrate are listed in Table 2.

### 2.5. Spectral Properties, Metal Content and Electron Paramagnetic Resonance (EPR) Analysis of Purified Melac13220

**Figure 7:**
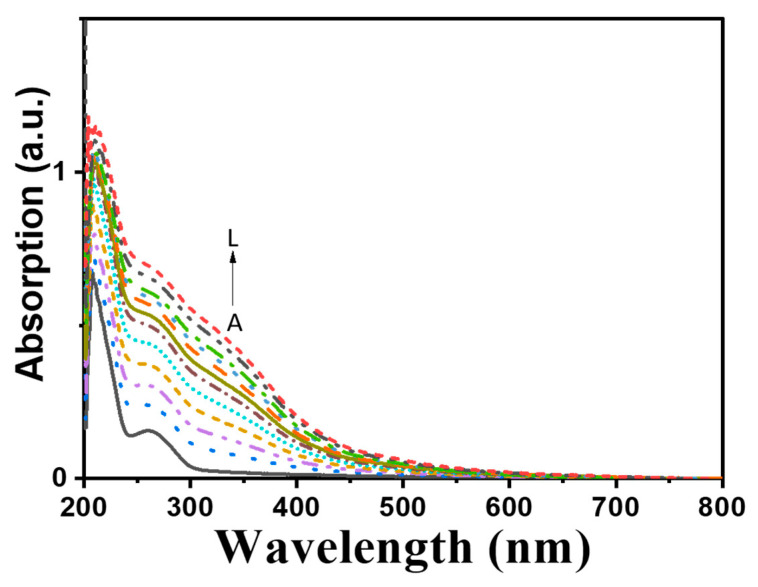
UV/visible absorption spectra of pure Melac13220 (0.6 U/mg) from in 20 mM Tris-HCl buffer (pH 7.5). The dotted lines show increasing Fe^2+^ concentrations from bottom to top (A–L, from 0.012 mM to 0.112 mM).

Full copper purified laccases exhibit an intense blue color and thus are also known as blue laccases [34]. In contrast to blue laccases, purified Melac13220 appeared as a colorless complex, and it demonstrated unique UV-visible absorption characteristics which were different from those of blue laccases. The spectra of purified laccase confirmed the absence of a visible peak near 610 nm, which was typical for T1 Cu. Simultaneously, there was no shouldering found at 330 nm corresponding to T3 binuclear copper, whereas a peak at 260 nm was detected (Figure 7).

The ICP-OES analysis demonstrated that the laccase derived from *Methylobacterium extorquens* showed 0.71 ± 0.03 copper ions and 2.33 ± 0.02 iron ions per protein molecule. The EPR spectrum was also silent; thus, no T1 and T2 Cu signals were detected (data not displayed). This may be due to the high electron content of Fe^2+^, which has a low spin state in protein molecules. The silence of EPR detection has also been observed in the “white” laccase POXA1 obtained from *Pleurotus ostreatus*, which contained one copper atom, one iron, and two zinc atoms [34], and in “white” laccase from *Phellinus ribis* [35], which contained only one copper atom, one manganese atom, and two zinc atoms in per molecule. In conclusion, the metal content in the active center and the total silencing observed for the UV/vis and EPR spectra indicate that the successfully purified “white” laccases were different from all the laccases reported to date.

### 2.6. Affinity of Fe^2+^ to Melac13220

**Figure 8:**
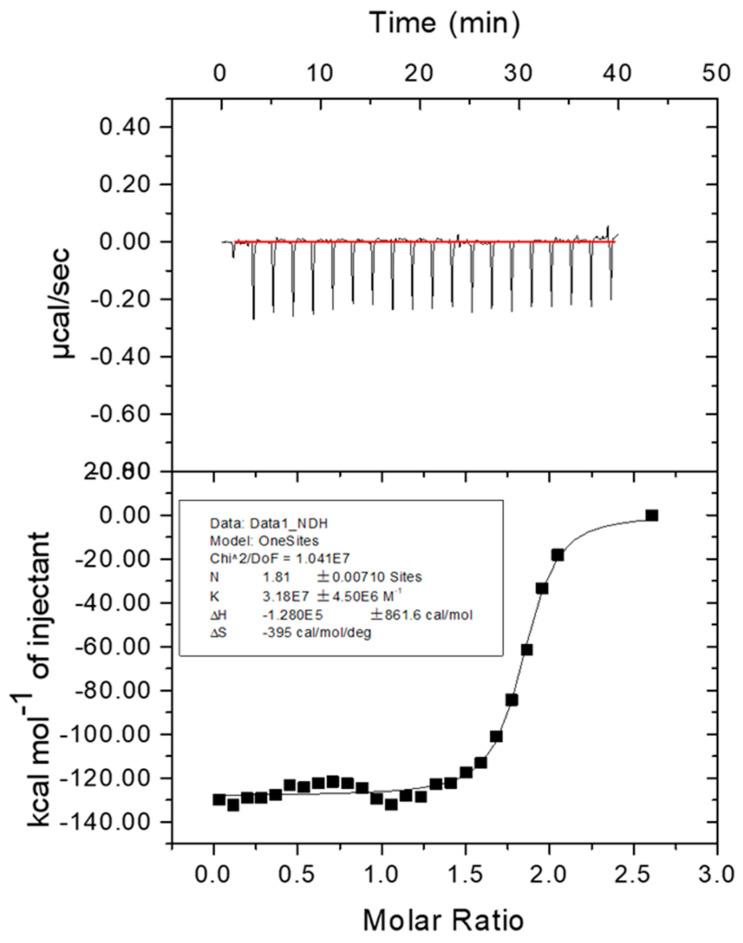
Isothermal titration calorimetry for the interaction of Fe^2+^ and Melac13220. Experiment conditions: C_Fe_^2+^ = 1 × 10^−4^ M, C Melac13220 = 6.3 × 10^−6^ M, T = 298 K.

Isothermal titration calorimetry (ITC) is considered an important means of explaining the binding mechanism. The typical ITC fitting curve is shown in Figure 8 with the measurement at 25 °C. The upper part of the figure shows the response value of heat absorption of the system after adding metal ions; the lower part of the graph shows binding parameters gained through one set of site modeling after the point-to-point deduction of the background with a typical ITC curve. With the formula (ΔG = ΔH − TΔS), ΔG < 0 was calculated. Moreover, we found that ΔH < 0 and ΔS < 0, indicating that the combination of ferrous ions and Melac13220 is a spontaneous process and that it is carried out at low temperatures. Additionally, the affinity output as a K was (3.18 ± 0.45) × 10^7^ M^−1^, and the number of binding sites was N = 1.81, which is approximately two, meaning that each Melac13220 molecule binds two ferrous ions. The thermodynamic parameters for the combination of Melac13220 and ferrous ions are shown in Figure 8.

**Figure 9:**
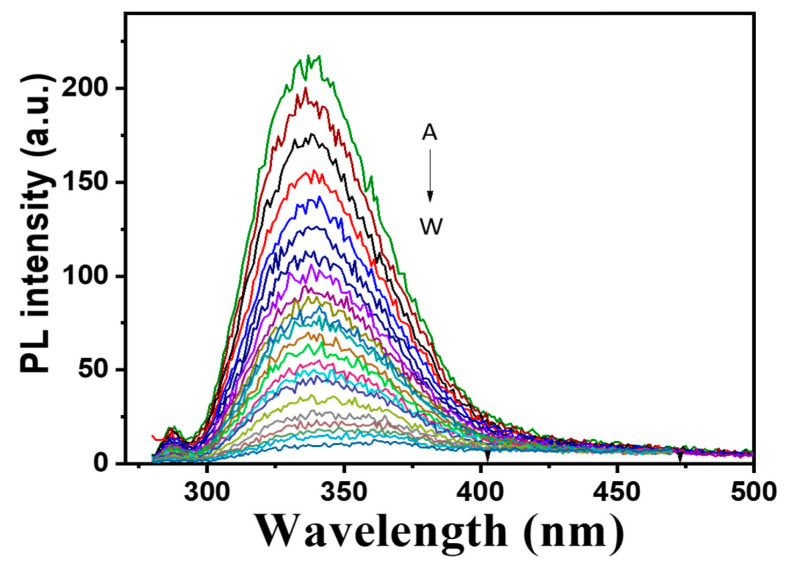
The photoluminescence spectra of Melac13220 laccase from *Methylobacterium extorquens* in 20 mM Tris-HCl buffer (pH 7.5). The dotted lines show the increasing Fe^2+^ concentration from top to bottom (A–W, from 0.01 mM to 0.47 mM).

Purified Melac13220 with different concentrations of Fe^2+^ (0.01~0.47 mM) was scanned in a photoluminescence spectrophotometer. The results are presented in Figure 9.

### 2.7. Decolorization of Dyes

The decolorizing efficiency of purified Melac13220 on various synthetic dyes was investigated to demonstrate its industrial applicability. Melac13220 (Table 3) was able to efficiently decolorize structurally different types of synthetic dyes, including azo and triphenylmethane dyes, without a laccase redox mediator. Melac13220 possessed an excellent decolorizing ability of over 90%, especially towards Congo Red. Table 4 summarizes the previous reports from other sources of laccases for the decolorizing of Congo Red dye.

## 3. Discussion

### 3.1. Enzymatic Characteristics of Melac13220

The ability of laccases to operate at higher temperatures is particularly important since elevated temperature often favors high reaction rates and substrate solubility in many processes, which is beneficial in decreasing enzyme load, thus reducing the risk of mesophilic microbial contamination, saving energy, and reducing the cost of the external cooling process [17].

In the characterization of laccase, the optimum pH value could vary with substrates and may be related to the oxidation mechanisms of different substrates, which may be caused by the difference in the redox potential of the laccase and the substrate [17]. With ABTS as a substrate, laccase activity can be detected in the lower pH range, which may be because ABTS exhibits nonphenolic properties [17]. The difference in redox potential between laccase and substrate is the driving force of the catalytic reaction. The potential difference of phenol substrate increased with the increase in pH, which can explain that the optimal pH of 2,6-DMP substrate is higher than that of ABTS substrate. Similar to our observations, some bacterial and fungal laccases have optimal pH values in the acidic range [38,39,40]. The changes in laccase activity and stability under different pH values were mainly caused by substrate specificity, the available oxygen, and differences in redox potential between the substrate and the enzyme.

### 3.2. Effect of Metal Ions (Fe^2+^) and Organic Solvent on Enzyme Activity

As a common environmental pollutant, metal ions are important factors that affect enzyme structure and function and affect the activity and stability of extracellular enzymes and the efficiency of enzymes in specific applications. Generally, heavy metal ions are effective inhibitors of enzyme reactions through the chelation of Cu (II) atoms or modification of the amino acid residues [28]. Studying the interaction between metal ions and laccase can help to better understand the biotechnological process of xenobiotic biodegradation [41]. For industrial applications, the enhanced activity of Melac13220 induced by Fe^2+^, Cu^2+^, and Co^2+^ may be a valuable property. The catalytic function of laccase in the presence of metal ions may be due to the interaction between metal ions and the copper center of the enzyme. The effect on laccase activity demonstrated by metal ions may be realized by chelating copper atoms or modifying protein structure through amino acid modification [42]. In most studies, laccases were reported to be strongly inhibited by Fe^2+^ [22,26,43]; here, we found that Fe^2+^ enhanced the enzyme activity of Melac13220 at low concentrations. There have been no other reports of Fe^2+^ enhancement of laccase activity. This will make up for the low enzyme activity of wild-type Melac13220 laccase, indicating that Melac13220 can be used in a much wider industrial field and in some specific reaction processes due to this property. When Fe^2+^ and Cu^2+^ act together, with a concentration under 1 mM, Fe^2+^ plays the dominant role, “pulling Cu^2+^ up”. When the concentration is above 1 mM, the activation role of Fe^2+^ is not so obvious. The differences in the activity of laccases derived from different sources in the presence of metal ions might be attributed to their origin and the type of metal ions used since both have a significant impact on the catalytic activity of the enzyme. Increasing laccase activity in the presence of heavy metal ions may be an advantage considering its wide use in many industrial fields such as pulp, paper, and bio-remediation. The activation or inhibition of proteolytic enzymes by metal ions may alter the turnover rate of extracellular enzymes [29]. A possible explanation for the reduced laccase activity in the presence of metal ions is that these ions adhere to type II and type III coppers and interfere with the internal electron transfer process, thereby affecting its activity. According to this assumption, the metal ions that make enzymes less active may react with the laccase’s electron-transferring system. The blockage of the substrate pathway or the transfer of electrons at the T1 site leads to an inhibition in enzyme activity [44]. Increased laccase activity in the presence of some metal ions, especially copper, has been observed for most laccases, and this can be interpreted as Cu^2+^ filling the type one Copper binding site [18,27,45]. 

As the most commonly used non-aqueous medium in biocatalysis, organic solvents affect the activity and stability of enzymes in many aspects. Studies have shown that the log *P* of organic solvents impacts the enzyme’s activity [33]. Through the MD simulation process, researchers have observed that acetonitrile molecules can penetrate into the active site of the enzyme, leading to structural changes that reduce its catalytic activity in aqueous solutions [33]. In addition, acetone can bind to some active sites on the enzyme and disturb its structure [33]. Melac13220 is a metalloenzyme, and the organic solvents tested above are both aerobic or nitrogen donors, which can directly coordinate with the active metal and inhibit the activity of enzymes. For example, DMSO interacts with or binds to the diiron site of monooxygenase during catalytic turnover [46]. Generally, lower catalytic activity results of enzymes in non-aqueous solutions can be attributed to diffusional limitations, restricted protein flexibility, low stabilization of the enzyme-substrate intermediate, the loss of water from protein structures, and the penetration of solvents into active sites [47]. Therefore, we can choose the synergy of solvent engineering and protein engineering as a potential strategy to improve the catalytic performance of enzymes in non-aqueous media [33]. In contrast, in some cases, enzymes perform well in organic solvents because substrate specificity, regional selectivity, and enantioselectivity could be modified by changing the organic solvent [47].

### 3.3. Substrate and Kinetic Analysis

Kumar et al. reported that K_m_ values of ABTS and 2,6-DMP were found to be 0.37 mM and 1.68 mM, respectively, for a non-blue laccase from *Pandoraea* sp. ISTKB [21]. However, the thermostable laccase from *Alcaligenes faecalis* XF1 (1243.75 s^−1^) demonstrated much higher catalysis efficiency than Melac13220 [18]. These differences might be due to their different active site geometries, which adapted to ABTS. Based on the results obtained when adding Fe^2+^ treatment under different substrates, the activation effect of Fe^2+^ on the enzyme was mainly evidenced by increasing the values of K_m_ and k_cat_ to different degrees. The catalytic functions of laccase in the presence of Fe^2+^ were probably involved with interactions between Fe^2+^ and the copper center of the enzyme. ABTS was the earliest synthetic mediator and the most commonly used laccase substrate mediator [48]. ABTS binds to the “pocket” of the enzyme primarily via hydrogen bonding, van der Waals interactions, and electrostatic forces [49]. However, the previous literature suggests that the oxidation efficiency and substrate affinity of laccase depend largely on the nature of the phenolic ring, the substitution of the phenolic ring, and the type of substrate used [18,50].

### 3.4. Spectral Properties of Purifies Melac13220

In general, Cu^2+^ with the d9 electron configuration creates a d-d transition and absorbs visible light under the action of ligands, and renders the blue color of a liquid [49]. Excluding contamination during the purification process, colorless proteins observed in this study may be caused by changes in the valence state of Cu^2+^. Therefore, the lack of absorption is most likely due to incomplete oxidation of copper, which makes the active center of the laccase prone to electron transfer, as it has a fully occupied d10 electron configuration, and there is no d-d transition that could occur might render the protein highly active [37]. This typical phenomenon was found in the presence of white laccase. In addition, the measured A_280_/A_610_ absorbance ratio of Melac13220 was 41, which was higher than most typical blue laccases (most of which presented values in the range of 15–30) [49] and similar to the results reported for white laccase [51]. As is evident from the photoluminescence spectra of Melac13220, fluorescence quenching occurred with the increase of the Fe^2+^ concentration. It is probable that N in the residues form Melac13220 formed a complex with Fe^2+^ and that the two transition electrons in the outer layer of N formed a co-orbital with Fe. The significant response of Fe^2+^ with N as the chelating atom may be due to the better thermodynamic affinity and faster chelation rate of iron ions, as well as their stronger oxidation, compared to that of other transition metal ions [52].

### 3.5. Decolorization of Synthetic Dyes

In most applications, many laccases are commonly used in conjunction with a redox mediator. However, the use of a redox mediator is expensive, and they have been shown to be toxic, thus causing more pollution. Dyes produced by the textile industry pose a great threat to environmental safety, and the role of fungal and bacterial laccases in industrial dye processing has been widely studied [23,27,53]. Laccase from *Pandoraea* sp. ISTKB effectively decolorizes Crystal Violet with more than 80% decolorization [21]. rLac from *Bacillus amyloliquefaciens* shows a considerably high decolorizing ability towards Remazol Brilliant Blue R [24]. Compared to the decolorization ability of Congo Red displayed by laccase in other studies, Melac13220 performed much better. For some structural dyes, Melac13220 is, therefore, considered to have great potential as a dye decolorizer than other laccases requiring a redox mediator.

## 4. Materials and Methods

### 4.1. Materials

The *Methylobacterium extorquens* strain was purchased from JCM Japan. Bacterial strains *Escherichia coli* DH5α, *Escherichia coli* BL21 (DE3), and plasmid pET-28a (+) were all kept in our laboratory. Pfu DNA polymerase and protein markers were ordered from TransGen Biotech (Beijing, China). T4 DNA ligase and restriction endonucleases were all purchased from Takara (Dalian, China). All kits, such as the Bacteria DNA Extraction Kit, Gel Extraction and Purification Kit, and Plasmid Mini Kit, were purchased from Bioteke (Wuxi, China). 2,2′-Azino-bis(3-ethylbenzothiazoline-6-sulfonate) (ABTS); 2,6-dimethylphenol (2,6-DMP), isopropyl-β-d-thiogalactopyranoside (IPTG), and the synthetic dyes Remazol Brilliant Blue R, Crystal Violet, Indigo Carmine, and Congo Red were commercially obtained from Sigma-Aldrich (St. Louis, MO, USA). All the solutions required for the experiment were prepared in deionized water, and reagents and chemicals with the highest purity level were used. Absorption spectra were recorded using SHIMADZU UV-2700 spectrophotometer (Shimadzu, Kyoto, Japan). TECAN infinite F200 Pro (Tecan, Männedorf, Swiss) was used for the protein assay.

### 4.2. Cloning, Expression, and Purification of Melac13220

The strain *Methylobacterium extorquens* was obtained from the Japanese Collection of Microorganisms (DSM 13060). A genomic kit extraction method was used to extract the DNA, and the laccase gene sequence was retrieved from the NCBI database (WP_147019975.1). The primer design software Primer 5.0 (Premier, Vancouver, BC, Canada) was used to design the primers as follows:

Sense primer: 5′ ATCTTACATATGATGCGAAATCCCGACCCG 3′ and the anti-sense primer: 5′ TGACACGGATCCTCAACTCACCTCGAAACT 3′. Underlined sequences refer to restriction sites of Nde I and BamH I, which were designed in the sense and anti-sense primers, respectively. The PCR amplification procedure was initiated at 95 °C for 5 min, followed by 30 cycles of 95 °C for 30 s, annealing at 53 °C for 30 s, extension at 72 °C for 3 min, and ending with 10 min at 72 °C. The DNA fragment was purified using a PCR purification Kit. The purified fragment was digested with Nde I and BamH I and inserted into the pET-28a (+) expression vector, which was digested with the same restriction enzyme. The construct was transformed into *E. coli* DH5α for gene cloning and plasmid propagation.

The constructed recombinant plasmid was transformed into *E. coli* BL21 (DE3) for protein expression after sequencing confirmation. Then, cultures were cultivated overnight in Luria-Bertani (LB) medium and incubated at 37 °C with 160 rpm shaking. The culture was incubated until the optical density (OD600) reached 0.6 under the above conditions, and 0.5 mM IPTG was added to induce the expression of Melac13220. The culture was supplemented with 1 mM CuSO4 and then further grown at 25 °C and 170 rpm for 8 h before harvesting by centrifugation at 9000× *g* at 4 °C. The collected cells were resuspended in Tris-HCl buffer (20 mM, pH 7.5) and subsequently disrupted via ultrasonication (2 cycles of 4 min each with a 1 min interval on ice water). Then, the supernatant and precipitate were separated via centrifugation at 13,000× *g* for 10 min.

The obtained supernatant was loaded onto an SP cationic exchange column (GE Healthcare), washed, and pre-equilibrated with Tris-HCl buffer (20 mM, pH 7.5) prior to use. The absorbed proteins were eluted with same buffer at the outset, and then a linear 0–1.0 M NaCl gradient solution in the same buffer was applied at 1 mL/min. The purity and molecular mass of the purified laccase were analyzed by 12% (*w*/*v*) sodium dodecyl sulfate-polyacrylamide gel electrophoresis (SDS-PAGE) and stained with Coomassie Brilliant Blue R250. Thereafter, the fractions with laccase were pooled and dialyzed overnight against 20 mM Tris-HCl buffer (pH 7.5), concentrated by PEG 20,000, and stored at −20 °C for further analysis.

### 4.3. Enzyme Assay

Laccase activity was determined spectrophotometrically using ABTS as a substrate. The ABTS substrate (ε_420 nm_ = 36,000 M^−1^ cm^−1^) produces ABTS radicals through two steps. First, a dark green ABTS +∙cation radial is generated, and second ABTS^2+^ forms through oxidation by laccase and shows absorbance at 420 nm [48]. The absorbance change is measured to determine whether the reaction occurred, and then it can be concluded whether the expressed laccase is active. The reaction mixture contained a suitable amount of the enzyme and 0.5 mM ABTS in 20 mM glycine-HCl buffer solution (pH = 1.5) for a total volume of 1.0 mL. The enzyme activity unit (U) was defined as the amount of enzyme required to catalyze the oxidation of 1.0 μmol of substrate per minute at 50 °C.

### 4.4. Effects of Temperature and pH on Laccase Activity and Stability

The optimal pH and temperature, as well as the effects of pH and temperature on laccase stability, were monitored via the oxidation of ABTS at 420 nm. The effect of temperature on enzyme activity was measured at different temperatures ranging from 30 °C to 90 °C with 10 °C increments using ABTS as the substrate. The relative enzyme activity was determined, with the highest activity set to 100%. The thermal stability of the enzyme was assessed by pre-incubating purified Melac13220 at various temperatures. At varying intervals, samples were withdrawn, and the activity of laccase without pre-incubation was set to 100%.

In order to determine the optimum pH, the reaction was measured by surveying activity at different pH values ranging from 1.0 to 4.0 in 20 mM glycine-HCl buffer (pH 1.0–3.0) and 20 mM acetic acid-sodium acetate buffer (pH 3.0–4.0) at increments of 0.5 using ABTS and 2,6-DMP as the substrate. The relative activity of the enzyme was calculated considering the maximum activity as 100%. The enzyme pH stability was detected by incubating laccase in different buffers with pH values from 1.0 to 10.5 at 50 °C for 1 h. As described above, samples were employed to measure residual activity, which was determined relative to initial activity (set as 100%) before incubation.

DSC was used to assess the thermal stability of the enzyme. The measurement was performed on a VP-DSC microcalorimeter (MicroCal, LLC, Northampton, MA, USA) in the temperature range of 30–90 °C with a heating rate of 1 °C per min, and each sample was scanned twice. Prior to measurement, the sample was degassed under vacuum and then loaded into a calorimetric cell. The DSC curve was corrected by subtracting a buffer-buffer scan as the instrument baseline. Routine data analysis within OriginTM was performed during DSC runs without regard to compromising real-time operations. The post-cycle thermostat was set to 25 °C, the pre-scan thermostat was set to 15 min, and the current pressure of the cells was maintained at 36 psi. Purified Melac13220 was diluted in Tris-HCl buffer (20 mM, pH 7.5) to a final concentration of 0.1 mg/mL for the protein scan. Before the protein scan, a buffer scan was obtained to subtract the substrate from the protein signal, and Origin 7.0 was used to fit the two-state model to determine the melting temperature of the enzyme.

### 4.5. Effects of Metal Ions and Organic Solvent on Melac13220

Various metal ions, including Fe^2+^, Cu^2+^, Co^2+^, Ni^2+^, Ba^2+^, Zn^2+^, Mg^2+^, Mn^2+^, and Ca^2+^ were used to evaluate the effects of metal ions on enzyme activity after pre-incubation with laccase (1 mg/mL) at 50 °C for 1 h. The concentration of metal ions in the system was 0.5 mM. ABTS was then added as a substrate, and the enzyme solution without any metal compound was used as a control to calculate the residual enzyme activity. The effect of various organic solvents on enzyme activity was investigated by adding each organic solvent to the enzyme solution (1 mg/mL) at 50 °C for 1 h. The concentration of organic solvent in the reaction system was 10% (*v*/*v*). The residual activity was calculated based on the control in the absence of any organic solvent, for which the enzyme activity was set to 100%.

### 4.6. Enzyme Kinetic Studies

For the enzyme kinetic studies, freshly purified laccases mixed with various concentrations of ABTS and 2,6-DMP (ε_468 nm_ = 49,600 M^−1^ cm^−1^) as the substrate (ranging from 0.15 to 0.4 mM) were measured at the optimal pH and at 50 °C, and the enzyme was present in excess during the reaction. The Origin 7.0 software (Northampton, MA, USA) was used to fit data to the Michaelis–Menten equation to calculate the estimates of the kinetic constants, namely, Michaelis–Menten constant (K_m_) and catalytic constant (k_cat_) values.

### 4.7. Spectral Characteristics, Metal Content and Electron Paramagnetic Resonance (EPR) Analysis of Melac13220

The UV- visible absorption spectra of purified enzymes before and after incubation with Fe^2+^ (0.012 mM–0.112 mM) was recorded using a UV-2700 UV/vis spectrophotometer (Shimadzu, Kyoto, Japan). All experiments were carried out at room temperature with 0.6 U/mg enzyme in 20 mM Tris-HCl buffer, pH 7.5.

The fluorescent measurements of the enzyme before and after incubation with Fe^2+^ (0.01–0.47 mM) were determined by RF-5301PC fluorescence spectrometer (Shimadzu, Kyoto, Japan). All experiments were carried out at room temperature with 0.6 U/mg enzyme in 20 mM Tris-HCl buffer, pH 7.5.

The metal content of purified enzyme was measured on an ICP-OES instrument (Agilent 725-ES, Agilent, Palo Alto, CA, USA). Protein samples (1 mg/mL) were mixed with ultra-pure nitric acid (67–70%) and nitrated at 200 °C for 15 min. After that, the nitrated sample was diluted to 50 mL with 2% nitric acid for metal content analysis.

Electron paramagnetic resonance (EPR) spectra of purified laccase were recorded by a Bruker Elexsys E500 spectrometer (Bruker, Karlsruhe, Germany) at 9.5 GHz, with a microwave power of 5.0 mW, a modulation frequency of 100 kHz, and amplitude modulation of 2 G. The sweep time used in the experiments was 120 s. The probe temperature was regulated by means of a liquid nitrogen cryostat equipped with a temperature control unit and kept at 100 K.

### 4.8. Affinity Analysis of Fe^2+^ to Melac13220

ITC (MicrocalTM iTC200, GE, Boston, MA, USA) was used to study the thermodynamics of biological interactions and binding between laccase and metal ions. A 0.1 mM Fe^2+^ solution and a 6.3 μM Melac13220 solution were prepared with 20 mM Tris-HCl buffer (pH 7.5). Prior to analysis, the Fe^2+^ solution was filtered through a 0.22 μm filter membrane, and 40 µL of the solution was drawn with a microprocessor-controlled motor-driven syringe titration needle on the pipette. The macromolecule pool (200 µL capacity) was filled with laccase solution, and the reference pool was filled with pure water using a gas-tight Hamilton syringe (500 µL capacity) with a thin stainless-steel capillary tube. The titration parameter reaction temperature was set to 25 °C, the total injection time was 20 drops, the reference power was 5 µCal/s, the initial balance interval was 60 s, the stirring speed was 1000 rpm, the volume of each drop was 0.4 µL, the time for a single injection was 0.8 s, the interval time of titration was 150 s, and the filter period time was 5 s. A control group was assessed to correct for the thermal effect of mixing and dilution by titrating buffer with Fe^2+^ solution. The Origin 9.0 software was used to process the raw experimental data.

### 4.9. Dye Decolorization by Melac13220

The dye decolorization ability of Melac13220 was evaluated by incubating the enzyme with various types of dyes, including azo dyes, such as Congo Red (λ_max_ = 488 nm); anthraquinoneic dyes, such as Remazol Brilliant Blue R (λ_max_ = 595 nm); and two triphenylmethane dyes, including Crystal Violet (λ_max_ = 590 nm) and Indigo Carmine (λ_max_ = 610 nm). The decolorization reactions using the purified Melac13220 were carried out in the dark at 50 °C for 24 h under mild shaking conditions. The 10.0 mL reaction mixture contained 80 mg/mL dyes in 20 mM Tris-HCl buffer (pH 7.5) for Congo Red and 20 mM Gly-HCl buffer (pH 1.5) for other dyes and 1.0 U/mL pure enzyme solution. The control group was a mixture of dye and buffer without enzyme, and the relative decrease in absorbance at 0, 10, and 24 h was measured by spectrophotometry. All experiments were carried out in triplicate.

## 5. Conclusions

In this study, the white laccase Melac13220 was successfully expressed in *E. coli* from the bacterium *Methylobacterium extorquens*, and a high-purity protein was obtained after one-step purification. The properties of the enzyme were then characterized: the maximum temperature of the enzyme was 65 °C. It maintained excellent stability over 40 °C and 50 °C, with a half-life of 50 h and 9 h, respectively. The optimal pH was 1.5, which makes this an eosinophilic enzyme that has high activity in acidic environments. It was also able to efficiently decolorize Congo Red and Indigo Carmine dyes.

According to the experimental results, Cu^2+^ and Co^2+^ slightly increased the enzyme activity, and Fe^2+^ was a strong activator of Melac13220 activity resulting in increases of up to five times or more. This phenomenon has not been previously reported. Since Melac13320 contains a Cu type one (T1) substrate-binding site, Fe^2+^ may affect the T1 active site, which influences enzyme activity [48].

Based on these characteristics of laccase Melac13220, it can be genetically modified to increase its stability and activity at high temperatures and to obtain highly active and stable enzymes to expand its range of application. The addition of Fe^2+^ in some reaction systems to increase enzyme activity and reaction speed and improve reaction efficiency can also be explored. The properties mentioned above render Melac13220 a promising substance for further industrial application. This study has laid an experimental basis for the application of this laccase in industrial and biological fields. Although this enzyme was a thermophilic enzyme with an optimum temperature of 65 °C, its activity was reduced to nothing because it was liable to aggregation after storage. Taking these issues into account, in our further study, we develop corresponding strategies to expand the industrial applications of this enzyme.

## Figures and Tables

**Table 1:** Kinetic parameters for Melac13220 obtained with ABTS and 2,6-DMP as reaction substrate before and after addition of Fe^2+^.

Substrate	K_m_ (mM)	k_cat_ (s^−1^)	k_cat_/K_m_ (mM^−1^ s^−1^)
ABTS ABTS (+Fe^2+^)	7.65 × 10^−2^ 9.76 × 10^−2^	14.6 74.5	190.85 763.32
2,6-DMP 2,6-DMP (+Fe^2+^)	9.18 × 10^−2^ 1.59 × 10^−1^	12.1 33.8	130.81 214.06

**Table 2:** Comparison of enzymatic parameters of different non-blue lac-cases.

Microorganism	Molecular Weight (kDa)	Optimum Temperature (°C)	Optimum pH	K_m_ (mM)	k_cat_ (s^−1^)	k_cat_/K_m_ (mM^−1^ s^−1^)	References
*Methylobacterium extorquens*	50	65	1.5	7.65 × 10^−2^	14.6	190.85	This study
*Pleurotus ostreatus*	61	60	7.0	9.0 × 10^−2^	5833	64811.1	[34]
*Phellinus ribis*	76	65	5.0	0.207	1333	6439.6	[35]
*Bacillus* sp. MSK-01	32	75	4.5	1.624	177	109	[36]
*Myrothecium verrucaria* NF-05	66	30	4.0	8.59 × 10^−2^	267.1	3109.4	[37]
*Trametes hirsuta*	90	85	2.4	7 × 10^−2^	197	2800	[9]

**Table 3:** Decolorization of the synthetic dyes using Melac13220; reaction mixture contained 1.0 U/mL of pure enzyme, 80 mg/mL of synthetic dyes in 20 mM Tris-HCl buffer (pH 7.5) for Congo Red and 20 mM Gly-HCl buffer (pH 1.5) for other dyes. The values represent the mean ± standard deviation of three independent measurements.

Dyes	λ_max_ (nm)	Decolorization (%)	
		10 h	24 h
Remazol Brilliant Blue R	595	13.9 ± 0.7	29.1 ± 1.3
Crystal Violet	590	10.7 ± 1.1	30.5 ± 1.5
Indigo Carmine	610	37 ± 0.6	54 ± 0.9
Congo Red	488	93 ± 1.2	99 ± 0.7

**Table 4:** Degradation of Congo Red using different sources of laccases.

Origin	Decolorization (%)		Reference
	10 h	24 h	
*Methylobacterium extorquens*	93%	99%	This study
*Pandoraea* sp. ISTKB	20%	10%	[21]
*Myrothecium verrucaria NF-05*	/	58%	[37]
*Trametes orientalis*	/	60%	[26]
*Cerrena* sp. HYB07	/	57%	[30]
*Alcaligenes faecalis XF1*	/	31%	[18]

## Data Availability

Not applicable.

## Supplementary Materials

The following supporting information can be downloaded at: https://www.mdpi.com/article/10.3390/ijms23179804/s1.
