# Supplementary material for: Characterization of a Novel Fe2+ Activated Non-Blue Laccase from Methylobacterium extorquens
Source: Int J Mol Sci. 2022 Aug 29;23(17):9804. doi: 10.3390/ijms23179804 (PMC9456135; doi:10.3390/ijms23179804)
Supplement: Supplementary file 1 [file ijms-23-09804-s001.zip › ijms-1859027-supplementary.pdf]

International Journal of
Molecular Sciences

MDPI

Article

Characterization of a Novel Fe^{2+} Activated Non-Blue Laccase from *Methylobacterium extorquens*

Abidan Ainiwaer, Yue Liang, Xiao Ye, Renjun Gao *

Key Laboratory for Molecular Enzymology and Engineering, The Ministry of Education, School of Life Science, Jilin University, Changchun 130012, China; abdan18@mails.jlu.edu.cn (A.A.); yue.liang@bofuconsulting.com (L.Y.); yexiao20@ mails.jlu.edu.cn (X.Y.)

* Correspondence: gaorj@jlu.edu.cn; Tel.: +86-431-18604313058; Fax: +86-431-85155200

The gene sequence of Melac13220

ATGCGAAATCCCGACCCGACGGCCCAGCCCGCGCCGGGATCGTTCTCACGGCGCGATCTGCTCGCGGGCA

GCGCCGCCCTCGCCCTGATGCCGCGGACGGCCGCGGCCCAAGCGCCGGCCGGGAAGGCCCCGCCCTCGGA

GCCGAAATCGATCAAGGCAGCACCGGCCACGCTCCGCCTGAAGCCCGAGCCCGCGCCTGAGACCGCCGTA

TGGCGTCTCGGCGAAGCGGAGGCACCGATCCTGCGGGTCAAGCTCGGCGAGGCAATCCGCCTGCGCGTCG

AGAACGGGACCGACAAGCCGCTCTCGCTGCACTGGCACGGGGTGCGGATCGTCAACGCCATGGACGGCGT

CGGCGGCGTCACCCAGGAGCCGATCAAGCCGGGGGCGAGCTTCACCTACGACTTCACGCCCCCGGATGCC

GGCAGCTTCCTGATCCGCGCGCTCGTTGTCGGCGGGTCGAGCGAGCCGTCGGGCCGGGGCATCGCCGGCA

TGCTGATCGTGGACGAGCCCTCCCCGCCCCCGGTCGATGCAGACCTCGCCCTGCTCGTGCAGGATTGGCG

GCTCGACGAGGCCGGCGCGCTGCAACCCTTCGGGCAGGTCGCCTTCGCCGCAGCGGCCGGGCGGCTCGGC

AGCGTCGTGACGCTCAACGGACGACCGATCCCGCTCGCACTCGACGCGCGGCCCGGCAGCCGTCTGCGCC

TGCGGCTCGCCAATGCCTGCAACGCGCGCAGCCTGCGCATCCGCTTCGATGGGCTGAAGGCTTATGTCGC

GGCCGTGGACGGGCAGCCGACCGACACGTTCGAGCCTCTGAAGGCGACACTCCCCTTCGCGCCGGGCACG

CGCTACGACCTGCTGCTCGATCTCCCGGCGCAGGCGGACCGGCCGGCACGATCACCGCGCTGGTCGGGC

AGGGCCTGCCGCTGGCGACCCTGACCGCCGTCGGCGAGCCGGTGGCGCAATCCGGCCGCGCCGCAATCGG

CTCGATCCCCGAGAACAAGCGCCTGCCGGCCGAGATCCGGTTGCAAAGCGCCCTTCGCCGCGAACTCGTG

CTGACGGGCGGCGTGCGGCCCGACAAGGCGAAGCCCGGCGCCGAGACGCCCTATTCCGGCGATCCCGCCA

AGATCTGGCAGATCAACGGCGCGAGCGGGACCGCGGGGGCGGCCCCCCTGTTCTCGGTCAAGCGCGGCGG

GGTCGTGGTGCTGTCGATCCGCAACGACACCGGCTTCATCCAGAGCCTGCACCTGCACGGCCACGTCTTC

CGCCTGCTGCACCAGCTCGATGACGGCTGGGAGCCCTACTGGCTCGACACCGTGCAGATCCCGGAAGGCA

AAAGCGCCCAGATTGCCTTCGTCGCGGACAATCCCGGCCGCTGGCTGCTCAGCGCGACCGTGCTGGAGCG

TTTCGATACCGGGCTATGGACGAGTTTCGAGGTGAGTTGA

Citation: Ainiwaer, A.; Liang, Y.; Ye, X.; Gao, R. Characterization of a Novel Fe^{2+} Activated Non-Blue Laccase from *Methylobacterium extorquens*. *Int. J. Mol. Sci.* **2022**, *23*, 9804. https://doi.org/10.3390/ijms23179804

Academic Editors: Zhou Cheng and Guimin Zhang

Received: 26 July 2022
Accepted: 25 August 2022
Published: 29 August 2022

Publisher's Note: MDPI stays neutral with regard to jurisdictional claims in published maps and institutional affiliations.

Copyright: © 2022 by the authors. Licensee MDPI, Basel, Switzerland. This article is an open access article distributed under the terms and conditions of the Creative Commons Attribution (CC BY) license (https://creativecommons.org/licenses/by/4.0/).

The protein sequence of Melac13220

1 MRNPDPTAQP APGSFSRREL FAGSAALALM PRTAAAQAPA

41 GKAPPSEPKS IKAAPATLRL KPEPAPETAV WRLGEAEAPI

81 LRVKLGEAIR LRVENGTDKP LSLHWHGVRI VNAMDGVGGV

121 TQEPIKPGAS FTYDFTPPDA GSFLIRALVV GGSSEPSGRG

161 IAGMLIVDEP SPPPVDADLA LLVQDWRLDE AGALQPFGQV

201 AFAAAAGRLG SVVTLNGRPI PLALDARPGS RLRLRLANAC

241 NARSLRIRFD GLKAYVAAVD GQPTDTFEPL KATLPFAPGT

281 RYDLLLDLPA QAGPAGTITA LVGQGLPLAT LTAVGEPVAQ

321 SGRAAIGSIP ENKRLPAEIR LQSALRRELV LTGGVRPDKA

361 KPGAETPYSG DPAKIWQING ASGTAGAAPL FSVKRGGVVV

401 LSIRNDTGFI QSLHLHGHVF RLLHQLDDGW EPYWLDTVQI

441 PEGKSAQIAF VADNPGRWLL SATVLERFDT GLWTSFEVS

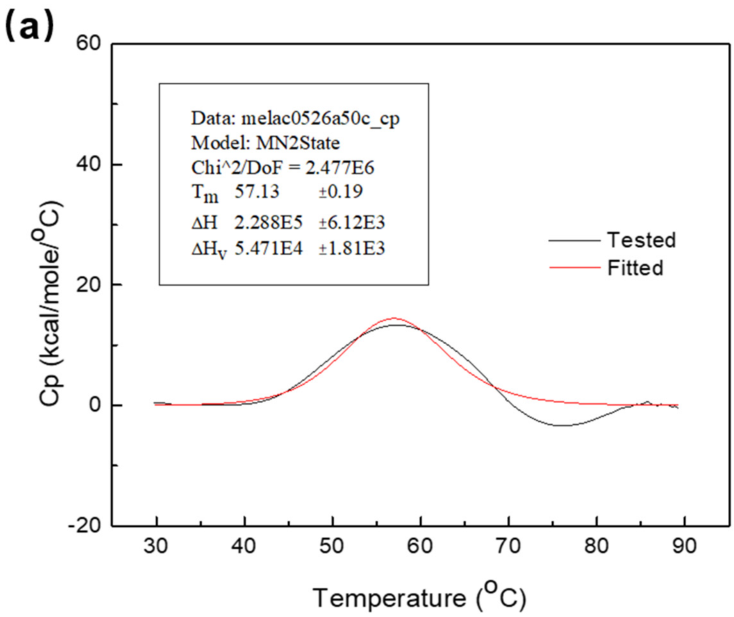

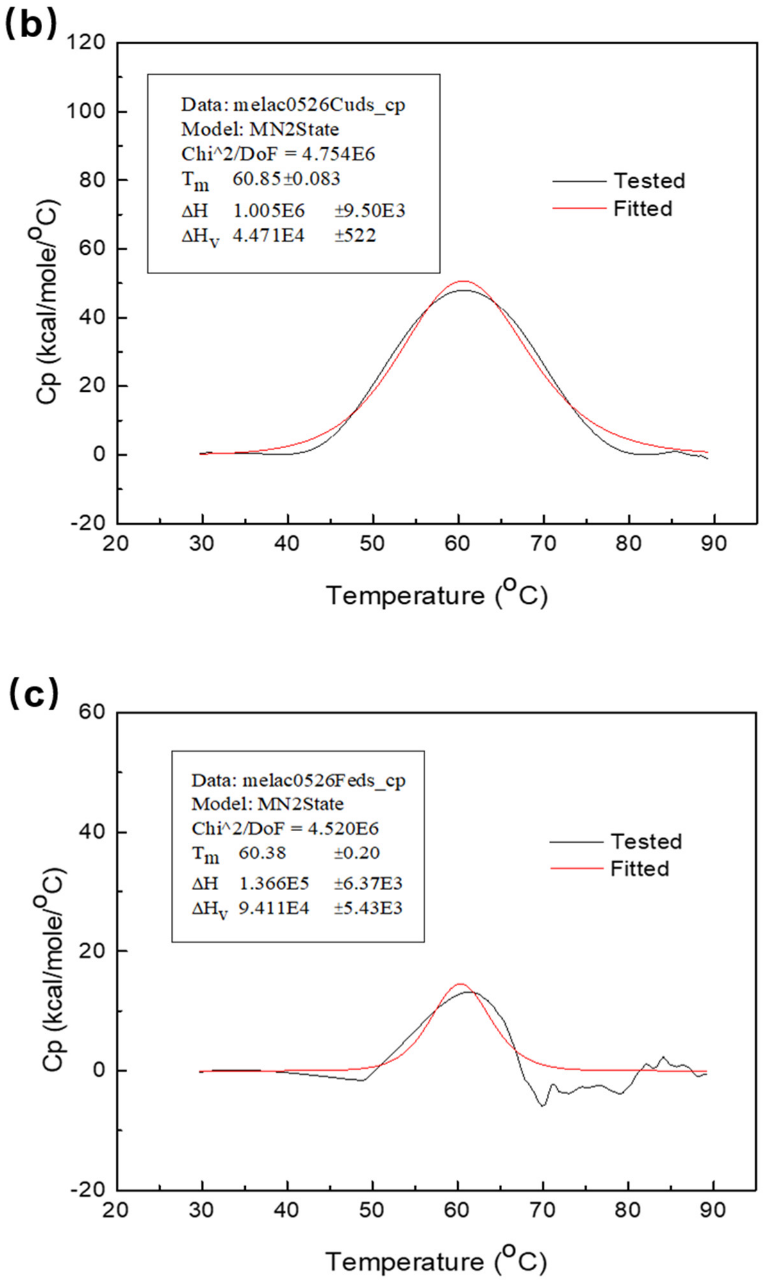

Figure S1. The curve of Differential Scanning Calorimetric. The melting temperature (T_m) and enthalpy of melting (ΔH_l) of purified Melac13220 (**a**), after adding Cu^{2+} (**b**), and Fe^{2+} (**c**).
